# Increased plasmablasts enhance T cell-mediated beta cell destruction and promote the development of type 1 diabetes

**DOI:** 10.1186/s10020-022-00447-y

**Published:** 2022-02-05

**Authors:** Qing Ling, Lei Shen, Wei Zhang, DuoDuo Qu, Hongdong Wang, Bin Wang, Yong Liu, Jing Lu, Dalong Zhu, Yan Bi

**Affiliations:** 1 Department of Endocrinology, Affiliated Drum Tower Hospital, Medical School of Nanjing University, Nanjing, China; 2 Shanghai Institute of Immunology, Shanghai Jiao Tong University School of Medicine, Shanghai, China; 3 Clinical Stem Cell Center, Affiliated Drum Tower Hospital, Medical School of Nanjing University, Nanjing, China; 4 Department of Laboratory Medicine, Affiliated Drum Tower Hospital, Medical School of Nanjing University, Nanjing, China

**Keywords:** Autoimmunity, B cell subset, Plasmablast, T cell, Type 1 diabetes

## Abstract

**Background:**

Although type 1 diabetes (T1D) is typically described as a T cell-mediated autoimmune disease, increasing evidence for a role of B cells has emerged. However, the pivotal disease-relevant B cell subset and its contribution to islet autoimmunity remain elusive.

**Methods:**

The frequencies and phenotypic characteristics of circulating B cell subsets were analyzed using flow cytometry in individuals with new-onset T1D, long-term T1D, type 2 diabetes, and nondiabetic controls, and also in a prospective cohort of patients receiving mesenchymal stromal cell (MSC) transplantation. NOD mice and adoptive transfer assay were used to dissect the role of the certain B cell subset in disease progression. An in-vitro coculture system of islets with immune cells was established to examine the response against islets and the underlying mechanisms.

**Results:**

We identified that plasmablasts, a B cell subset at the antibody-secreting stage, were significantly increased and correlated with the deterioration of beta cell function in patients with new-onset T1D. Further, a fall of plasmablast number was associated with the preservation of beta cell function in patients who received MSC transplantation after 3 months of follow-up. Meanwhile, a gradual increase of plasmablasts in pancreatic lymph nodes during the natural progression of insulitis was observed in non-obese diabetic (NOD) mice; adoptive transfer of plasmablasts together with T cells from NOD mice accelerated diabetes onset in NOD/SCID recipients.

**Conclusions:**

Our study revealed that plasmablasts may function as antigen-presenting cells and promote the activation and proinflammatory response of CD4^+^ T cells, further contributing to the T cell-mediated beta cell destruction. Our results provide insights into the pathogenic role of plasmablasts in islet autoimmunity and may offer new translational strategies for inhibiting T1D development.

**Supplementary Information:**

The online version contains supplementary material available at 10.1186/s10020-022-00447-y.

## Background

Type 1 diabetes (T1D) is an autoimmune disorder characterized by the progressive deterioration of pancreatic beta cell function, insulin deficiency, and hyperglycemia (Barnett 2018). The pathogenesis of T1D involves islet infiltration by various components of immune systems (insulitis), which results in ongoing islet autoimmunity and destruction of beta cells. Although autoreactive CD8^+^ T cells and CD4^+^ T cells are classically considered as the primary effectors mediating beta cell damage, other immune cells are also crucial for disease development (Boldison and Wong 2016).

B cells are typically considered as the main source of autoantibodies against specific beta cell proteins of glutamate decarboxylase (GADA), tyrosine phosphatase (IA2A), and zinc transporter 8 et al., which serve as hallmarks of T1D onset (Ling et al. 2018; Lampasona and Liberati 2016). However, emerging evidence also indicates an active role of B cells directly involved in beta cell destruction. Studies have demonstrated that B cells are required for the development of diabetes in non-obese diabetic (NOD) mice, which can be prevented by B cell depletion (Smith et al. 2017; Hu et al. 2007; Serreze et al. 1996; Falcone et al. 1998). B cell depletion therapy with an anti-CD20 antibody in new-onset patients with T1D showed efficacy in preserving beta cell function for 1 year; whereas such therapy showed limited effect after 2 years (Pescovitz et al. 2014; Pescovitz et al. 2009). Thereafter, little progress has been made until recently, studies of human pancreas from T1D donors draw attention to the role of B cells in islet autoimmunity (Wang et al. 2019; Damond et al. 2019; Leete et al. 2016). B cells were especially enriched in the proximal region of islets with remnant beta cells, which were linked to a rapid beta cell loss in young patients (Wang et al. 2019; Leete et al. 2016). Meanwhile, increasing studies begin to focus on abnormalities of certain B cell subsets. Decreased regulatory B cells and intolerance of antigen-specific B cells have been reported to participate in T1D progression (Smith et al. 2018; Smith et al. 2015; Kleffel et al. 2015). Hence, an in-depth analysis of B cell subsets in T1D progression is important for understanding the disease pathogenesis and determining optimal immunotherapies.

Up to now, only a few studies have examined the profile of B cell subpopulations in T1D; moreover, such studies apply distinct strategies for cell detection. One study analyzed B cell subsets based on their localization in second lymphoid tissues (Deng et al. 2016). Other studies stratified peripheral B cells as naïve, unswitched memory B cells, switched memory B cells, plasmablasts, and plasma cells, representing different stages during differentiation (Parackova et al. 2017; Hanley, et al. 2017; Thompson et al. 2014; Viisanen et al. 2017; Yang et al. 2019). Nevertheless, these studies fail to provide consistent results on the alteration of B cell subsets. Furthermore, the mechanisms underlying altered B cell subsets and their role in promoting T1D progression still remain elusive. Therefore, more efforts are required to clarify the pivotal disease-relevant B cell subset and its associated mechanisms contributing to T1D pathogenesis.

In this study, we focused on characterizing the major altered B cell subset associated with the development of T1D. By analyzing alterations in circulating B cell subsets from patients with T1D, we found plasmablasts were obviously elevated and associated with the deterioration of beta cell function. Moreover, in our recently published immunotherapy using mesenchymal stromal cells (MSC) in patients with T1D, clinical remission characterized by improved beta cell function was significantly improved after MSC transplantation. By analyzing the change of plasmablasts in recipients during MSC therapy, the association between decreased plasmablasts and preservation of beta cell function was demonstrated. The characterization and pathological effects of plasmablasts were further investigated in NOD mice which highlight their key role in the autoimmune response to pancreatic beta cells.

## Materials and methods

### Participants

Cross-sectional cohorts of 36 patients with new-onset T1D, 20 with long-term T1D, 22 with new-onset type 2 diabetes, and 26 sex-matched nondiabetic control subjects were recruited during 2017–2019 at the Endocrinology Department of Affiliated Drum Tower Hospital, Medical School of Nanjing University. Diabetes was diagnosed according to American Diabetes Association criteria (American Diabetes Association 2020). Patients with T1D were positive for at least one islet autoantibody and/or a fasting C-peptide ≤ 200 pmol/L. Time from diagnosis was categorized as < 1 year for new-onset and ≥ 1 year for long-term diabetes. The sex-matched type 2 diabetes patients were recently diagnosed and were seronegative for islet autoantibodies. The control individuals were euglycemia and without islet autoantibodies. Exclusion criteria were: malignancy; allergic state; acute infection in the past 3 months; other autoimmune diseases, e.g., rheumatoid arthritis and systemic lupus erythematosus; pregnancy; history of immune suppressive medication for more than 7 days.

In our prospective cohort study of MSC transplantation in T1D (ChiCTR2100045434), fourteen participants with available peripheral blood mononuclear cells (PBMC) samples were included in this study to analyze the change of plasmablasts during immunotherapy. Details about the MSC therapy were described as previously reported (Lu et al. 2021). Briefly, MSCs were isolated from Wharton’s Jelly of umbilical cord using the tissue explant method in GMP-certified facilities and underwent a variety of qualification test. Patients were given 1 × 10^6^ cells/kg as one single intravenous infusion and were followed up after 3 months. Responders were defined as patients with a 10% increase from baseline in the level of fasting and/or postprandial C-peptide.

### Laboratory test

Standard-meal tolerance test was performed for beta cell function evaluation. After a 10 h overnight fast, each participant received steamed bread with 2.5 g/kg for child and 100 g (containing 75 g carbohydrates) for adult. The levels of plasma glucose and C-peptide before and 2 h after food stimulation were analyzed. HbA1c levels were recorded. GADA, IA2A, and insulin antibodies (IAA) were detected by ELISA; and islet cell antibodies (ICA) was detected by indirect immunofluorescence (all from Oumeng, Beijing, China).

### Flow cytometry

**Fig. 1:**
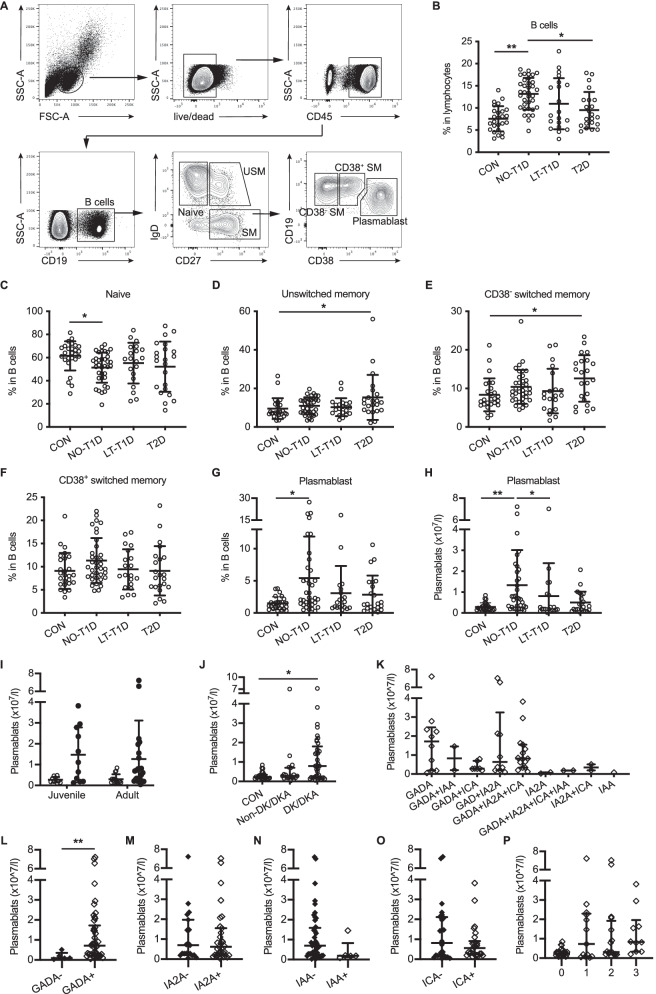
Circulating plasmablast frequency is increased in patients with new-onset T1D. **A** Flow cytometry analysis of circulating B cell subsets. **B** The frequency of B cells in control subjects (n = 26), NO-T1D patients (n = 36), LT-T1D patients (n = 20), and T2D patients (n = 22). **C**–**G** Frequencies of naïve B cells, unswitched memory B cells, CD38^−^ switched memory B cells, CD38^+^ switched memory B cells, and plasmablasts. One-way ANOVA followed by Bonferroni’s multiple comparisons test or Kruskal–Wallis test followed by Dunn’s multiple comparisons for plasmablast analysis and One-way ANOVA followed by Bonferroni’s multiple comparisons test for other subsets. **H** The absolute number of plasmablasts. * *P* < 0.05, ** *P* < 0.01. **I** Numbers of plasmablasts between patients with NO-T1D (filled-in circles) and controls (open circles) in juveniles (CON n = 10, T1D n = 11) and adults (CON n = 15, T1D n = 24). **J** Numbers of plasmablasts in the control subjects, NO-T1D patients with ketosis or ketoacidosis at onset, and NO-T1D patients without ketosis or ketoacidosis at onset. **K**–**O** Numbers of plasmablasts in patients seroconverting to different types of autoantibodies. **P** Numbers of plasmablasts in patients with different numbers of autoantibodies. Independent *t* test or Mann–Whitney *U* test. * *P* < 0.05, ** *P* < 0.01. Error bars indicate SD. CON, controls; NO-T1D, new-onset T1D; LT-T1D, long-term T1D

Human PBMCs were isolated using a BD Mononuclear Cell Preparation Tube according to manufacturer’s instruction. Samples were stained with fluorochrome-conjunct antibodies specific for various cell markers (Additional file 1: Table S1). Fixable Viability Stain 780 was used to discriminate viable cells. Data were collected with an LSRFortessa flow cytometer (BD Biosciences, Franklin Lakes, NJ, USA). According to the process of differentiation after antigen stimulation, B cell subsets were identified as: naïve (CD19^+^IgD^+^CD27^−^), unswitched memory (CD19^+^IgD^+^CD27^+^), CD38^−^ switched memory (CD19^+^IgD^−^CD27^+^CD38^−^), CD38^+^ switched memory (CD19^+^IgD^−^CD27^+^CD38^+^), and plasmablasts (CD19^+^IgD^−^CD27^+^CD38^hi^). The gating strategy is shown in Fig. 1A.

For mouse samples, cells prepared from spleens and lymph nodes were stained with anti-CD138, anti-CD44, anti-CD3e, anti-CD4, and anti-CD8. Plasmablast in mice was characterized by dual staining of CD138 and CD44 (Matsumoto et al. 2014) (Additional file 1: Fig. S1). For intracellular staining, cells were incubated with Leukocyte Activation Cocktail with BD GolgiPlug for 4–6 h and then processed with Fixation/Permeabilization Solution Kit and stained for IFN-γ, TNF-α (BioLegend), granzyme B (eBiosciences), and perforin (eBiosciences) (both from San Diego, CA, USA). Cells were analyzed or sorted with a FACSAria III cell sorter (BD Biosciences). Reagents were purchased from BD Biosciences unless otherwise indicated. Data were analyzed with FlowJo version 10.0 software (Tree Star, Ashland, OR, USA).

### Mice

Female NOD/ShiLtJ mice and NOD/SCID mice (NOD background, impaired T and B lymphocyte development, deficient natural killer cell function) were obtained from Model Animal Research Center of Nanjing University (Nanjing, China) and housed in a specific-pathogen-free environment (12-h light/dark cycle) with food and water ad libitum. The NOD/ShiLtJ mouse is a model of T1D. NOD/SCID mice were used for adoptive transfer experiments.

### Adoptive transfer

Plasmablasts and CD3^+^ T cells were purified (purity > 95%) from spleens and pancreatic lymph nodes (PLNs) from NOD mice (≥ 16-week-old) by FACSAria III. NOD/SCID mice (6-week-old) were injected intravenously with 2 × 10^6^ T cells, 3–5 × 10^5^ plasmablasts, or with a mixture of 2 × 10^6^ T cells and 3–5 × 10^5^ plasmablasts. Controls were treated with PBS. Glucose levels were monitored at least twice weekly using a FreeStyle Blood Glucose Monitoring System (Abbott Diabetes Care Ltd., USA). Diabetes were defined as two consecutive readings ≥ 13.9 mmol/L (250 mg/dL) (Tan et al. 2018; Zhang et al. 2020).

### Histological analysis

Pancreas were harvest into 4% paraformaldehyde and embedded in paraffin. Tissue Sections (3 μm) were stained with H&E (Servicebio, Wuhan, China) or processed for staining for insulin (GB11334, Servicebio). Insulitis was scored on the basis of islet infiltration with the following grades: grade 0, no insulitis or peri-insulitis; grade 1, insulitis involving < 25% islet; grade 2, insulitis involving 25–50% islet; grade 3, insulitis involving 50–75% islet, and grade 4, insulitis involving > 75% and/or complete islet infiltration (Akazawa et al. 2015). More than 100 islets were scored in each group. Images were taken using Pannoramic MIDI (3DHISTECH Ltd., Budapest, Hungary).

Immune cells infiltrating into the islets were detected by immunofluorescence. Tissue sections were processed for triple-staining with anti-CD138 (ab34164, Abcam, Cambridge, UK), anti-insulin (83506S), and anti-CD4 (GB13121) or anti-CD8 (GB13429) (all from Servicebio) after EDTA antigen retrieval. The cell nuclei were visualized with DAPI (G1012, Servicebio). Images were captured by an ECLIPSE C1 Microscopy and a DS-U3 digital camera (both from Nikon, Japan).

### Co-culture of mouse islets with immune cells

Islets were isolated from 4-week-old NOD mice according to published protocols (Han et al. 2001; Bleich et al. 1999). Briefly, 1 mL cold Hank’s balanced salt solution containing type XI collagenase (1 mg/mL, sigma) was infused into the bile duct via catheter. The inflated pancreas was removed, minced, and digested in a shaker-type water bath at 37 °C. Islets were picked by hand under a microscope and were cultured together in RPMI 1640 medium (glucose: 11.1 mmol/L) containing 10% FBS, 100 units/mL penicillin, and 100 mg/mL streptomycin at 37 °C in a humidified 5% CO_2_ atmosphere overnight.

Islets were then cultured in the 96-well round bottom plate (20–30/well) with mIL-2 (10 ng/mL) under conditions: (1) islets alone (control group); (2) with CD4^+^ T cells (2 × 10^5^/well); (3) with plasmablasts (1 × 10^5^/well); (4) with CD4^+^ T cells (2 × 10^5^/well) and plasmablasts (1 × 10^5^/well). CD4^+^ T cells and plasmablasts were sorted from spleens and PLNs of 16-week-old NOD mice. For blocking experiments, plasmablasts were pretreated with neutralizing anti-MHC class II mAb (2.5ug/mL, eBiosciences) or IgG isotype control antibody (2.5ug/mL, eBiosciences) for 1 h before cocultured with islets. After 4 days, supernatants were analyzed with a Luminex assay (LXSAMSM, R&D systems) following the manufacturer’s protocol. Islets were collected for apoptosis analysis using PE Annexin V Apoptosis Detection Kit I (BD Bioscience).

### Statistics

SPSS version 23.0 (IBM Corporation, Chicago, IL, USA) and GraphPad Prism 8 (GraphPad Software, San Diego, CA, USA) were used for statistical analyses. Student’s *t* test (for normally distributed data) or Mann–Whitney *U* test (for skewed data) was used for comparisons between two groups, whereas one-way ANOVA (for normally distributed data) or Kruskal–Wallis test (for skewed data) followed by post hoc comparisons was used for three or more groups. Differences in categorical variables were determined by the χ2 analysis or Fisher exact test. Correlations were performed using Pearson or Spearman correlation analysis. Diabetes incidence data were plotted as Kaplan–Meier curves and analyzed using the log-rank test. Two-tailed *P* < 0.05 was considered statistically significant.

## Results

### Circulating plasmablast frequency is increased in patients with new-onset T1D and associated with GADA

We recruited patients with new-onset T1D (n = 36), long-term T1D (n = 20), type 2 diabetes (n = 22), and healthy subjects (n = 26). Table 1 summarizes the features of the subjects in each group. There was no difference in age, gender, and BMI between the control, new-onset T1D, and long-term T1D groups. We analyzed B cells using flow cytometry and characterized the major altered B cell subpopulation in peripheral blood based on the differentiation states. The frequencies of total B cells (CD19^+^) were obviously increased in the new-onset T1D, long-term T1D, as well as type 2 diabetes groups (Fig. 1A and B). Furthermore, no significant difference was observed in the frequencies of CD38^+^ switched memory, CD38^−^ switched memory, and unswitched memory B cells in patients with new-onset T1D when compared with the controls and the type 2 diabetes group, while the proportion of naïve B cells was decreased in the new-onset T1D group relative to the controls (Fig. 1C to F). The most notable difference was observed in the plasmablast subset which was significantly increased in patients with new-onset T1D compared with the controls; however, patients with long-term T1D and type 2 diabetes showed no obvious change in the proportion and the number of plasmablasts (Fig. 1G and H).

**Table 1:** Characteristics of participants

	CON (n = 26)	NO-T1D (n = 36)	LT-T1D (n = 20)	T2D (n = 22)	*P* value
Gender, Men (n)					
Age (years)	22.2 ± 9.3	26.1 ± 14.2	27.9 ± 15.3	47.3 ± 12.2^a,b,c^	< 0.001
< 18 years, n (%)	10 (38)	12 (33)	6 (30)	0 (0)	
≥ 18 years, n (%)	16 (62)	24 (67)	14 (70)	22 (100)	
Onset-age (years)	/	26.1 ± 14.3	24.0 ± 15.6	47.1 ± 12.8^b,c^	< 0.001
Duration (months)	/	2.0 ± 2.1	45.8 ± 23.5	1.3 ± 1.3^c^	< 0.001
BMI (kg/m^2^)	20.8 ± 2.5	19.5 ± 3.0	20.6 ± 3.3	24.0 ± 2.5^a,b,c^	< 0.001
DK or DKA (%)	0.0	75.0	55.0	9.1	< 0.001
Family History of Diabetes (%)	30.8	25.0	15.0	54.5	0.033
HbA1c (mmol/mol)	34 ± 3.3	84 ± 31.7	65 ± 26.2	96 ± 31.7	
HbA1c (%)	5.3 ± 0.3	9.8 ± 2.9^a^	8.1 ± 2.4 ^a^	10.9 ± 2.9^a,c^	< 0.001
FPG (mmol/l)	4.3 ± 0.5	7.4 ± 2.8^a^	10.1 ± 4.8 ^a,b^	9.5 ± 3.4^a,b^	< 0.001
2hPG (mmol/l)	4.5 ± 1.3	13.5 ± 4.6^a^	13.3 ± 3.6 ^a^	15.8 ± 3.6^a^	< 0.001
FCP (pmol/l)	558.4 (489.2, 719.0)	226.0 (141.4, 323.2)^a^	32.3 (9.8, 242.3)^a^	520.4 (348.5, 789.1)^b,c^	< 0.001
2hCP (pmol/l)	1717 (1321.0, 2261.0)	425.5 (243.2, 795.8)^a^	82.9 (16.5, 387.0)^a^	964.9 (747.61782.0)^b,c^	< 0.001
IAA^+^	0	2	3	0	
ICA^+^	0	22	5	0	
GADA^+^	0	34	13	0	
IA2A^+^	0	24	8	0	
No. of autoantibodies					
4		1	1		
3		15	1		
2		13	8		
1		7	5		

Data are n, %, mean ± SD, and median (25th–75th percentile). /, not appropriate. ^a^P < 0.05 vs CON; ^b^P < 0.05 vs NO-T1D; ^c^P < 0.05 vs LT-T1D; NO-T1D, new-onset T1D; LT-T1D, long-term T1D; T2D, type 2 diabetes; FPG, fasting plasma glucose; 2hPG, 2 h postprandial plasma glucose; FCP, fasting C-peptide; 2hCP, 2-h postprandial C-peptide; DK, diabetic ketosis; DKA, diabetic ketoacidosis

Further, we examined alterations of plasmablasts in T1D with heterogeneous traits. Patients with new-onset T1D showed increased numbers of plasmablasts compared with their age-matched controls in both juveniles and adults (Fig. 1I). Moreover, plasmablast number was increased in patients complicated by diabetes ketosis or ketoacidosis at onset, but not in those without this emergency (Fig. 1J). Additionally, we analyzed the number of plasmablasts in patients with multiple islet autoantibody positive (Fig. 1K). The significant elevation of plasmablasts was observed in patients who were positive for GADA (Fig. 1L) but not in those with IA2A, ICA, or IAA positive (Fig. 1M to O). There was no significant difference in plasmablast number in patients with different numbers of autoantibodies (Fig. 1P).

### The increase of circulating plasmablasts correlates with the deterioration of beta cell function in T1D

**Fig. 2:**
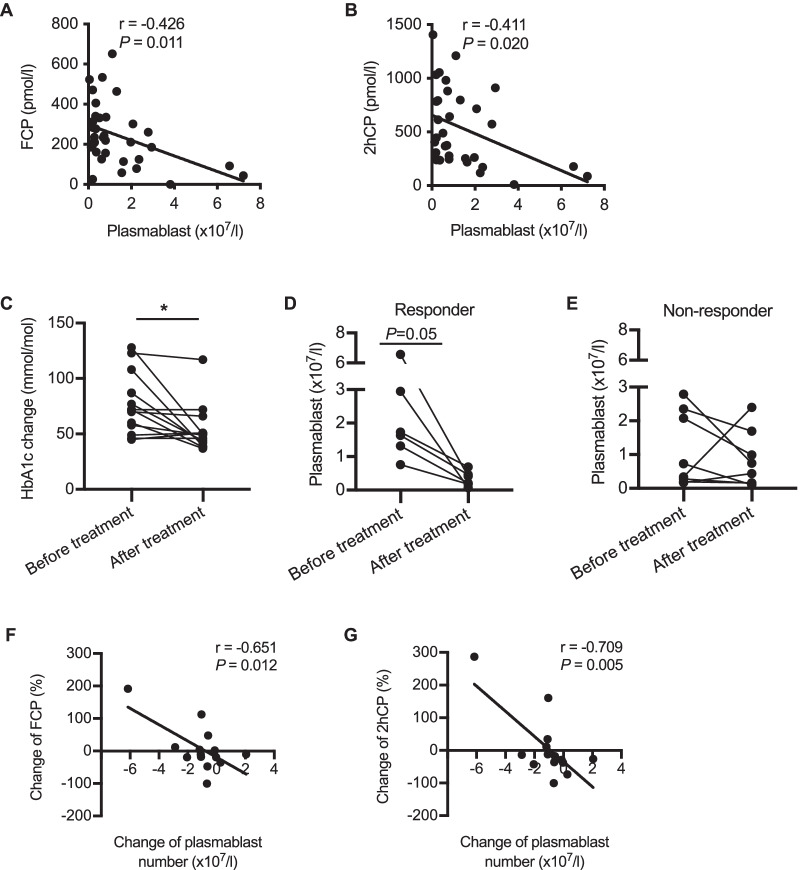
The increase of plasmablasts correlates with the deterioration of beta cell function. **A**–**B** Correlation between the number of plasmablasts and FCP (n = 35) and 2hCP (n = 32) in patients with new-onset T1D. **C** HbA1c levels in patients receiving MSC transplantation at baseline and 3 months after transplantation. **D**–**E** Plasmablast number at baseline and 3 months after treatment in responders (**D**) and non-responders (**E**). **F**–**G** Correlation between the altered plasmablast number and the change of FCP and 2hCP. Correlation was assessed by Pearson analysis. Difference between the data before and after MSC treatment were assessed by Paired *t* test. * *P* < 0.05. FCP, plasma fasting C-peptide; 2hCP, plasma 2-h postprandial C-peptide

To explore the potential role of increased plasmablasts in beta cell destruction, we analyzed the relationship between plasmablast number and beta cell function in patients with new-onset T1D. Meaningfully, the number of plasmablasts was negatively associated with fasting C-peptide (r = − 0.426, *P* = 0.011), as well as 2 h postprandial C-peptide (r = − 0.411, *P* = 0.020) in patients with new-onset T1D (Fig. 2A and B).

After verifying their eligibility as well as informed consent, 14 patients with new-onset T1D received MSC transplantation. Three months after transplantation, the level of HbA1c was significantly decreased (Fig. 2C, P < 0.01). The responders (6/14) displayed reduced plasmablast numbers after MSC transplantation, while the non-responders showed inconsistent changes of plasmablasts (Fig. 2D and E). Importantly, both the change of fasting C-peptide (r = − 0.651, *P* = 0.012) and 2 h postprandial C-peptide (r = − 0.709, *P* = 0.005) were negatively correlated with the altered number of circulating plasmablasts (Fig. 2F and G). Taken together, these results indicate a close relationship between the increase of plasmablasts and the deterioration of beta cell function.

### Circulating plasmablasts in T1D retain the feature of antigen presentation

**Fig. 3:**
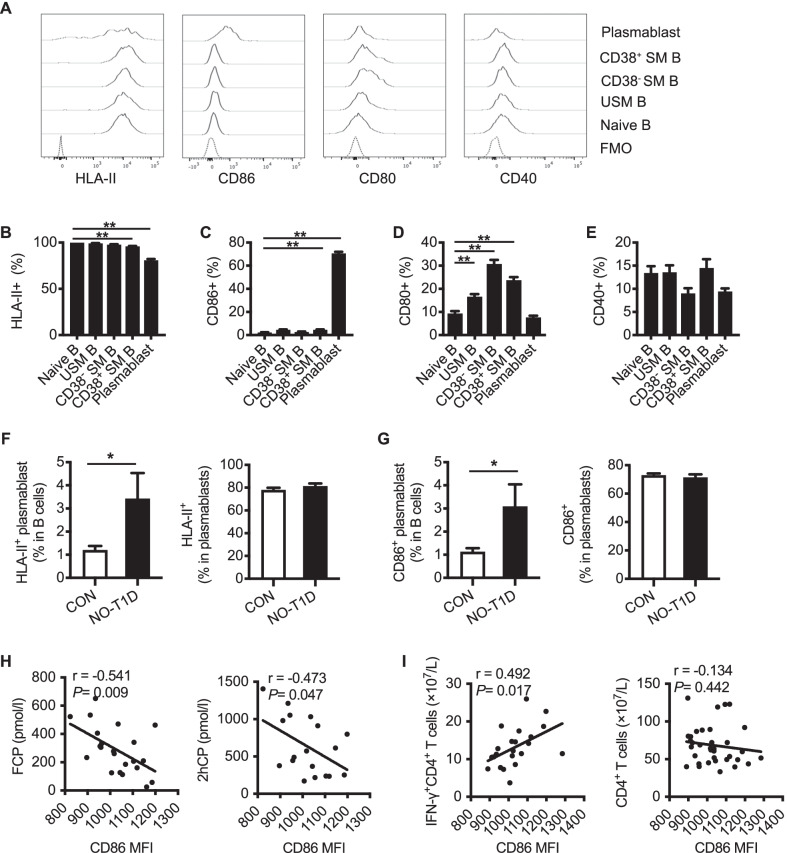
Plasmablasts in T1D retain the antigen-presenting feature. **A** Representative histograms of HLA-II, CD86, CD80, and CD40 expression on B cell subsets from patients with new-onset T1D. **B**–**E** The analyses of the HLA-II, CD86, CD80, and CD40 expression on B cell subsets. Data indicate mean ± SEM. Difference between groups were assessed by One-way ANOVA followed by Dunnett's multiple comparisons test. ** *P* < 0.01 compared with naïve B cells. **F**–**G** Comparison of the HLA-II^+^ and CD86^+^ plasmablast frequencies in B cell compartment between control subjects and patients with new-onset T1D. Data indicate mean ± SEM. Independent *t* test, * *P* < 0.05. **H** Association between CD86 expression and FCP and 2hCP in patients with new-onset T1D. **I** Association between CD86 expression on plasmablasts and IFN-γ^+^CD4^+^ T cells in patients with new-onset T1D. Correlation was assessed by Pearson analysis. FCP, plasma fasting C-peptide; 2hCP, plasma 2-h postprandial C-peptide

B cells have been previously reported to present beta cell-specific antigens and activate diabetogenic T cells (Smith et al. 2017). As a differentiated subset of B cells, plasmablasts are well known for their antibody secretion capacity. Interestingly, by analyzing the expression of HLA-II, CD86, CD80, and CD40 on circulating B cell subsets (Fig. 3A to E), we found that about 80% of plasmablasts retained HLA-II expression (Fig. 3B), and CD86 expression was much higher on plasmablasts than naïve B cells (Fig. 3C). Further, compared with control subjects, patients with new-onset T1D displayed significantly higher proportions of HLA-II^+^ and CD86^+^ plasmablasts among B cells (Fig. 3F and G), but no difference in the proportion of plasmablasts expressing CD80^+^ or CD40^+^ (Additional file 1: Fig. S2). Additionally, the CD86 expression on plasmablasts negatively correlated with fasting C-peptide (r = − 0.541, *P* = 0.009) and 2 h postprandial C-peptide (r = − 0.473, *P* = 0.047) (Fig. 3H), and positively correlated with IFN-γ^+^CD4^+^ T cell counts (r = 0.492, *P* = 0.017) in new-onset T1D subjects, whereas not with that of the overall CD4^+^ T cells (Fig. 3I). These results suggest plasmablasts retain the antigen-presenting feature which was closely associated with the development of T1D.

### Plasmablasts are increased in the spleen, PLNs, and islets with T1D progression in NOD mice

**Fig. 4:**
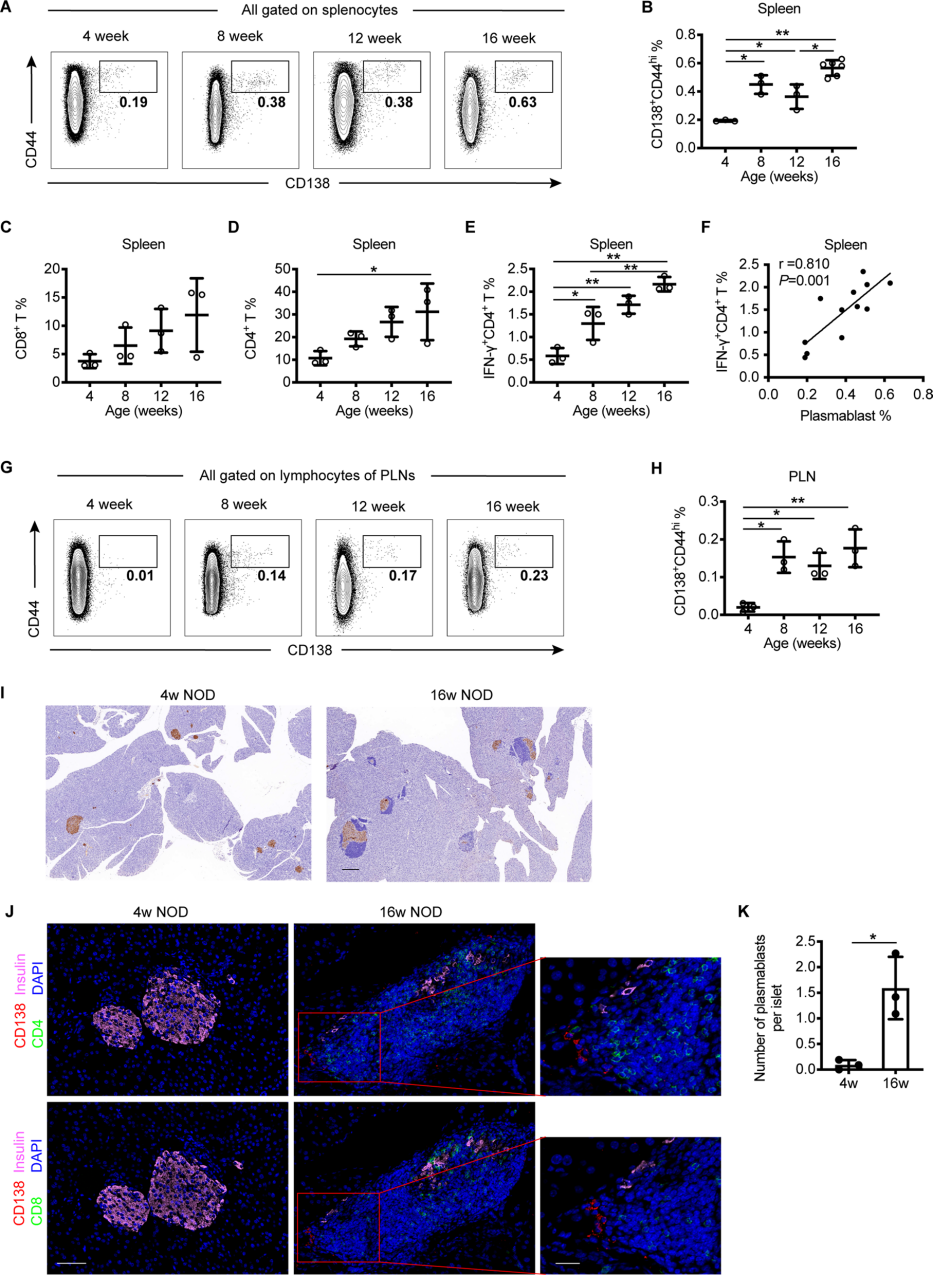
Plasmablasts are increased with the progression of T1D in NOD mice. **A**–**B** Representative plots and frequencies of plasmablasts in the spleens of NOD mice at 4, 8, 12 and 16 weeks of age. **C**–**E** The frequencies of CD8^+^ T cells, CD4^+^ T cells, and IFN-γ^+^CD4^+^ T cells among splenocytes of NOD mice at 4, 8, 12 and 16 weeks of age. **F** Association between the frequencies of plasmablasts and IFN-γ^+^CD4^+^ T cells among splenocytes in NOD mice at different age. **G**–**H** Representative plots and the frequencies of plasmablasts in the PLNs of NOD mice at 4, 8, 12 and 16 weeks of age. Data indicated mean ± SD. Difference were analyzed by One-way ANOVA followed by Bonferroni’s multiple comparisons test. Correlation was assessed by Pearson analysis. * *P* < 0.05, ** *P* < 0.01. **I** Representative immunohistochemical staining for insulin (brown) in pancreatic islets from 4- and 16-week-old NOD mice. Scale bar, 200 μm. **J** Representative immunofluorescence staining of pancreatic islets from 4- and 16-week-old NOD mice. Pancreatic specimens were co-stained for CD138 (red), CD4 or CD8 (green), insulin (pink) and dapi (blue). Scale bar, 50 μm (left and middle), 20 μm (right, represent the boxed area in the middle). **K** Quantification of plasmablasts in pancreatic islets from 4- and 16-week-old NOD mice (n = 3 per group, a total of 56 islets in 4-week-old group and 59 islets in 16-week-old group were analyzed)

To figure out the expansion process of plasmablasts during the natural history of T1D, we measured plasmablasts in the spleen, PLNs, and islets from NOD mice at 4, 8, 12, and 16 weeks of age. Plasmablasts were dramatically increased in the spleens of NOD mice at 8, 12, and 16 weeks of age compared with 4-week-old mice (Fig. 4A and B). Similarly, CD4^+^ and CD8^+^ T cell subsets showed a trend toward increase during disease progression, with a significant increase of IFN-γ^+^CD4^+^ T cells in an age-dependent manner (Fig. 4C to E). Concordantly, the frequency of plasmablasts was positively associated with that of IFN-γ^+^CD4^+^ T cells (Fig. 4F).

The PLN is a critical site for the presentation of islet-associated autoantigens to self-reactive T cells. We found that plasmablasts were increased in the PLNs of NOD mice at 8, 12, and 16 weeks of age compared with 4-week-old mice (Fig. 4G and H). Islets from 4-week-old NOD mice displayed well-preserved islet structures with abundant insulin staining, while as the disease progressed, islets exhibited substantial lymphocyte infiltration in 16-week-old NOD mice (Fig. 4I). Immunofluorescence analysis revealed significantly more plasmablasts infiltrating into the islets of NOD mice at 16 weeks compared with those at 4 weeks of age and co-localized with CD4^+^ and CD8^+^ T cells around islets (Fig. 4J and K and Additional file 1: Fig. S6). These results indicate the abnormal increase and islet-infiltration of plasmablasts along with T cells are associated with T1D progression.

### Adoptive transfer of plasmablasts promote the onset of T1D in NOD/SCID mice

**Fig. 5:**
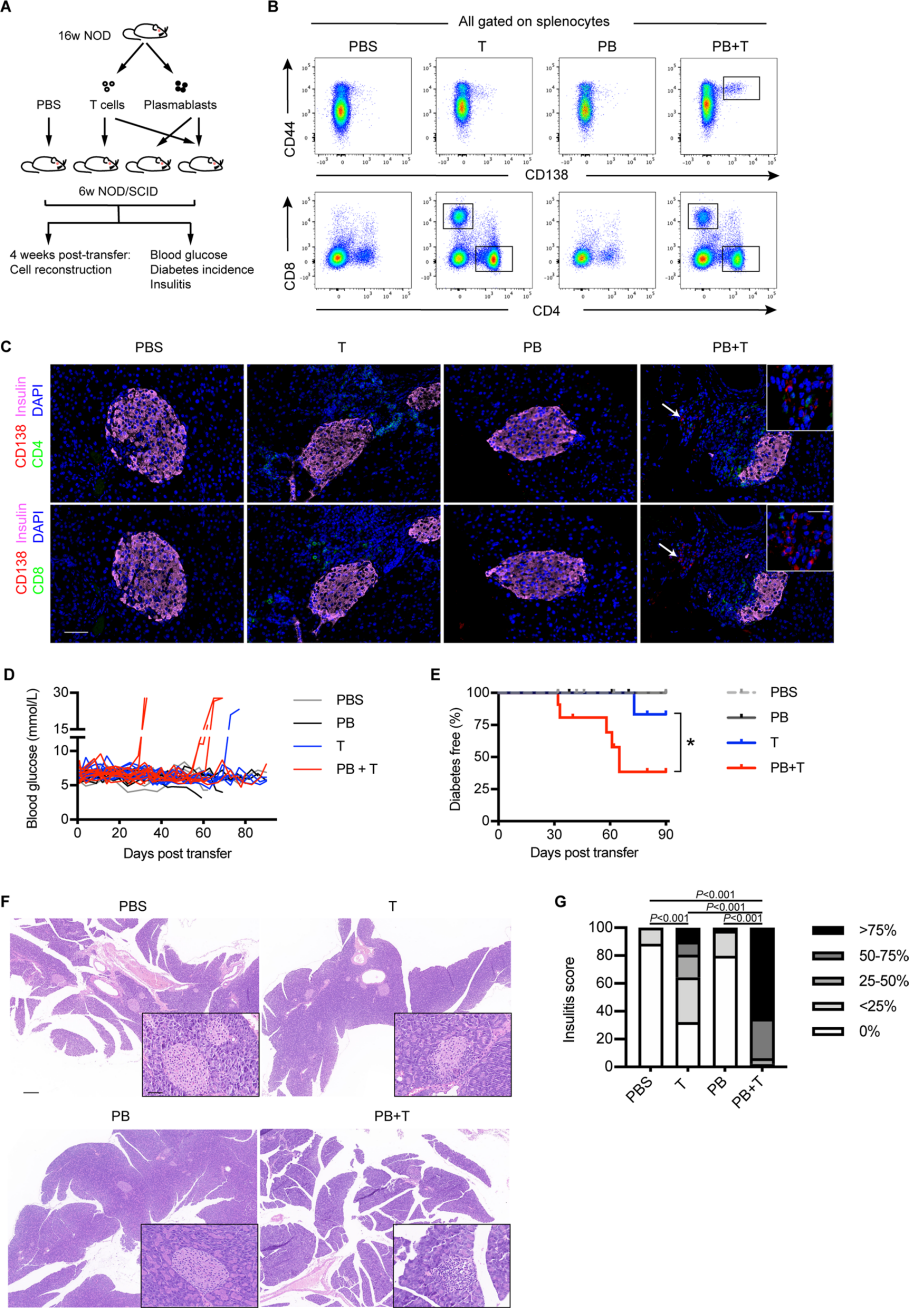
Adoptive transfer of plasmablasts accelerates the onset of T1D in NOD/SCID mice. **A** Schematic of the adoptive transfer experiment. **B** Representative plots indicating the reconstruction of adoptively transferred cells in the spleen of recipient mice 4 weeks after transfer. **C** Representative images indicating the staining of plasmablasts (CD138^+^, red) and CD4^+^ or CD8^+^ T cells (green) in the pancreatic islets from recipients. Insulin, pink. Dapi, blue. Scale bar: 50 μm; 20 μm (inset). **D** Blood glucose levels of recipient mice. **E** Diabetes incidence of recipient mice. Figure D&E show the pooled data of two independent experiments. Log-rank test. *P < 0.05. **F** Representative H&E staining of pancreas from recipient mice. Scale bar: 200 μm; 50 μm (inset). **G** A bar graph showing insulitis severity in indicated groups. Chi-square test. These data are representative of two independent experiments

To further clarify the causative role of plasmablasts in T1D development, we adoptively transferred T cells (T group, n = 11), plasmablasts (PB group, n = 6), or plasmablasts combined with T cells (PB + T group, n = 11) into 6-week-old NOD/SCID mice (euglycemia), respectively. NOD/SCID mice that received PBS injection were taken as controls (CON group, n = 7) (Fig. 5A). One month after transfer, recipient mice in PB + T group showed significantly accumulated plasmablasts in the spleens, PLNs, and islets, while recipients in other groups did not (Fig. 5B and C and Additional file 1: Fig. S3 and Fig. S7).

Meaningfully, mice in the PB + T group developed diabetes faster than those in the T group (Fig. 5D). After 90 days, 45.5% (5/11) of mice in the PB + T group and 9.1% (1/11) of mice in the T group developed diabetes respectively, whereas mice in the PB group and CON group remained normoglycemia (Fig. 5E P = 0.02). Histological analysis of pancreatic sections showed that normal islet architecture was lost in recipient mice in the PB + T group, which was replaced by a large number of immune cell infiltration (Fig. 5F). Most islets in other groups were free of insulitis or with minimal infiltration in the form of peri-insulitis. Recipient mice in the PB + T group showed the highest insulitis score among the four groups (Fig. 5G, P < 0.001). These findings indicate that plasmablasts may play a role in accelerating the T cell-mediated T1D development.

**Fig. 6:**
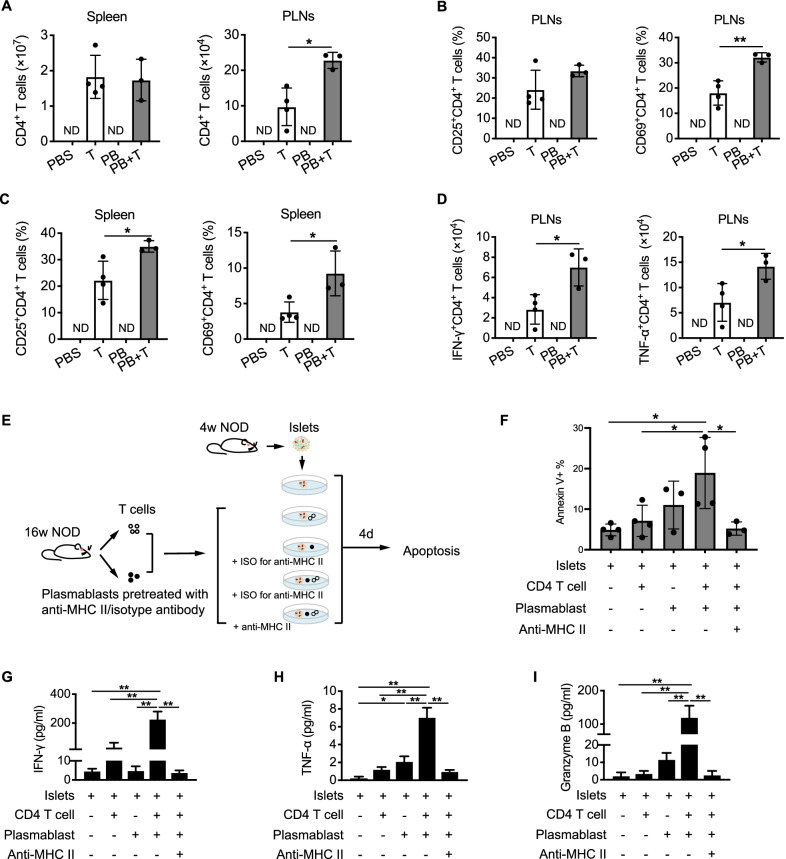
Plasmablasts promote T cell-mediated beta cell apoptosis through antigen presentation. **A** Comparison of CD4^+^ T cell numbers in the spleens and PLNs between PB + T group and T group. **B**–**C** Comparison of activated CD4^+^ T cells expressing CD25 or CD69 in the PLNs (**B**) and spleens (**C**) between PB + T group and T group. **D** Comparison of IFN-γ^+^CD4^+^ T cells and TNF-α^+^CD4^+^ T cells in the PLNs between PB + T group and T group. Data indicated mean ± SD. Difference were analyzed by Independent *t* test. * *P* < 0.05, ** *P* < 0.01. ND indicated not determined. **E** Schematic diagram of the co-culture experiment as described in the methods. **F** Apoptosis of islet cells in the indicated conditions. **G**–**I** Levels of IFN-γ (**G**), TNF-α (**H**), and granzyme B (**I**) in the supernatants of the indicated conditions. Data indicated mean ± SD. Difference were analyzed by One-way ANOVA followed by Bonferroni’s multiple comparisons test. * *P* < 0.05, ** *P* < 0.01. Data indicated mean ± SD. One-way ANOVA followed by Tukey’s multiple comparisons test. * *P* < 0.05, ** *P* < 0.01

Further, we examined the activation and intracellular cytokines of T cells in the recipient mice. The total number of CD4^+^ T cells in the spleens of PB + T group and T group were comparable, but significantly more CD4^+^ T cells were observed in the PLNs of PB + T group than T group (Fig. 6A). Additionally, CD4^+^ T cells from mice in PB + T group showed a higher frequency of activated CD69^+^ population in the PLNs, as well as higher expressions of activation markers of CD25 and CD69 in the spleen (Fig. 6B and C). Proinflammatory cytokines of IFN-γ and TNF-α were highly expressed by CD4^+^ T cells in the PLNs of the PB + T group relative to the T group (Fig. 6D). However, neither the proliferation nor the activation of CD8^+^ T cells in the spleens or PLNs showed any difference between PB + T group and T group (Additional file 1: Fig. S4A to D). Together, these data suggest that the promotion of diabetes progress by plasmablasts may be associated with the enhanced activation and proinflammatory response of CD4^+^ T cells.

### Plasmablasts promote T cell-mediated beta cell apoptosis through antigen presentation

To characterize mechanisms underlying the synergetic response between plasmablasts and CD4^+^ T cells during immune destruction of islets, we established an in vitro co-culture system of islets with immune cells (Fig. 6E). Co-culture of plasmablasts combined with CD4^+^ T cells resulted in a significantly increased beta cell apoptosis relative to the control group, whereas no difference was observed in those co-cultured with CD4^+^ T cells or plasmablasts alone (Fig. 6F). Further, the supernatant from the co-culture of islets together with plasmablasts and CD4^+^ T cells contained much higher levels of IFN-γ, TNF-α, and granzyme B compared with other culture conditions (Fig. 6G to I). These results indicate that plasmablasts promote islet beta cell destruction by facilitating the proinflammatory response of CD4^+^ T cells.

We next investigated whether plasmablasts induced the proinflammatory CD4^+^ T cells through antigen presentation. After pretreatment with neutralizing anti-MHC class II mAb, the amounts of proinflammatory cytokines were obviously reduced (Fig. 6G to I). Consistently, the addition of anti-MHC II mAb resulted in a significant attenuation in the cytotoxic effect of co-cultured plasmablasts and CD4^+^ T cells on islet beta cells (Fig. 6F). Therefore, our findings suggest that plasmablasts activate the diabetogenic CD4^+^ T cells via presenting islet autoantigens, resulting in the exacerbation of beta cell destruction.

## Discussion

Increasing evidence suggests a vital role for B cells participating in T1D progression. However, altered B cell subpopulations and the mechanisms by which contribute to T1D pathogenesis remain elusive. The present study reveals major alterations in the peripheral B cell compartment in patients with T1D. Plasmablasts are specifically increased in patients with new-onset T1D and correlated with the deterioration of beta cell function. Further, we provide evidence for a role of plasmablasts in aggravating insulitis and promoting disease onset, yet depending on a synergistic effect with T cells. The mechanism underlying plasmablast-promoted beta cell destruction is at least partially through autoantigen presentation to CD4^+^ T cells.

Studies attempting to characterize T1D-relevant alteration in the B cell compartment showed inconsistent results. Similar to our finding, Parackova et al. reported a skewed peripheral development of B cells in T1D younger than 20 years old, with a shift towards plasmablasts and a decrease in the transitional B cells (Parackova et al. 2017). But others did not find any disturbance in the peripheral B cell compartment (Hanley et al. 2017; Thompson et al. 2014; Viisanen et al. 2017). The discrepancy may result from variations in study cohorts, cell markers for detection, and measuring techniques. In our study, plasmablasts increased in individuals with new-onset T1D but did not in those with longer duration, indicating that plasmablasts may participate more in the onset of the disease. Besides, participants with relatively larger sample size and with different T1D endotypes were recruited, which implied that plasmablast expansion correlated to the hyperglycemic crisis at diagnosis and the positive islet autoantibody of GADA, indicating an involvement of plasmablasts in islet autoimmunity.

Increased plasmablasts in T1D may either play a role in beta cell destruction or serve as a biomarker of disease status. The former role is supported by both our observational analyses in the new-onset T1D and prospective analysis in the MSC-intervention cohort which showed a decreased number of plasmablasts along with preserved beta cell function. Therefore, plasmablasts are probably part of the imbalanced immune network promoting beta cell destruction. Besides their pathological role in the autoimmune response, the increase of circulating plasmablasts may serve as an indicator for the development of T1D, as supported by their potential to be a biomarker of disease activity in other autoimmune disorders (Banchereau et al. 2016; Pozdzik et al. 2016). Thus, the sensitivity or specificity of circulating plasmablast for evaluation of T1D trajectory may deserve further cohort studies.

To verify our findings in humans, we investigated the role of plasmablasts during the natural disease course in NOD mice. As insulitis progressed, plasmablasts were increased in spleens and concomitantly accumulated in target tissues of PLNs and islets. In agreement with our results, previous studies reported that B cells infiltrating the islets of NOD mice contained a high proportion of plasmablast- or plasma cell-like subpopulation (Serreze et al. 2011; Boldison et al. 2019). These studies further pointed out a potential relationship between the infiltration of this subpopulation and the limited efficiency of B cell-targeted therapy in preventing or reversing T1D (Serreze et al. 2011; Boldison et al. 2019). The authors used an anti-CD20 antibody to deplete B cells in NOD mice and found that plasmablast-like cells repopulated pancreatic islets after treatment; moreover, these cells with a plasma cell-like phenotype were probably resistant to anti-CD20. Thus, the recruitment of plasmablasts in islets probably exert a pathogenic role in beta cell destruction.

We next provided evidence for the potential role of plasmablasts in T1D development and revealed their pathological effect via interaction with T cells. In our clinical samples of new-onset T1D, a correlation between the activation of plasmablasts and CD4^+^ T cells was observed. Consistently, during the natural progression of insulitis in NOD mice, both the number and the activation of CD4^+^ T cells were increased in parallel with plasmablast expansion, indicating that there may be an interaction between plasmablasts and CD4^+^ T cells. Such a potential interaction and its effect on islets were demonstrated in an in vitro co-culture system, which showed that co-culture of islets with a mixture of plasmablasts and CD4^+^ T cells induced more severe beta cell apoptosis than co-culture with each of the two alone. As a further indication in adoptive transfer assays, co-transfer of plasmablasts and T cells, rather than plasmablasts alone, accelerated the disease onset in NOD/SCID mice, supporting that the pathological effect of plasmablasts is T cell-dependent. Therefore, there is likely to be a synergistic effect between plasmablasts and T cells in T1D pathogenesis.

Previous studies have elucidated the biological characteristics of plasmablasts, including producing protective and/or pathogenic antibodies, secreting cytokines, and in some conditions expressing antigen-presenting molecules as well (Nutt et al. 2015; Tiburzy et al. 2014). In T1D, plasmablasts are traditionally considered as antibody-secreting cells and mainly involved in producing the hallmarks of T1D. But their pathogenic role in mediating autoimmune destruction of islets remains unraveled. In our study, the phenotype and the function of plasmablasts were characterized as the following: (1) abundant expression of antigen-presenting proteins (HLA-II and CD86) with a negative association between CD86 expression and beta cell function; (2) association of increased plasmablasts and beta cell damage during the progression of T1D in both patients and mice; (3) synergistic effect of plasmablasts and T cells in accelerating T1D onset and in promoting beta cell apoptosis. Therefore, our findings addressed the pathogenic function of plasmablasts other than producing autoantibodies. Plasmablasts may directly activate diabetogenic T cells via presenting beta cell autoantigens, which in turn mediates beta cell damage.

Although the antigen-presentation function of antibody-secreting cells was previously reported in a physiological manner, our study firstly clarified the pathological contribution of plasmablasts in islet autoimmunity. A previous study reported plasma cells, a terminally differentiated subset similar to plasmablasts, expressed molecules for antigen presentation and pointed out their physiological function in preventing immune overreaction (Pelletier et al. 2010). In our co-culture system of islets with CD4^+^ T cells, however, plasmablasts facilitate the T cell reactions against beta cells. Notably, the administration of MHC-II blocking antibodies attenuated the beta cell apoptosis and production of proinflammatory cytokines by CD4^+^ T cells. Therefore, these data further confirm the antigen-presenting process of plasmablasts. Of note, the TNF-α secretion increased and the apoptosis of beta cells were slightly induced when plasmablasts alone co-cultured with islets (Fig. 6F and H). It needs further study whether plasmablasts affect the apoptosis of islet beta cells in other ways independent of T cells, such as producing inflammatory cytokines or pathogenic autoantibodies.

There are several limitations. Firstly, this study lacks investigation into other mechanisms by which plasmablasts contribute to T1D progression. Plasmablasts have been shown to express IL-17, IL-10, TGF-β, and function as cellular modulators in infection or autoimmune diseases (Matsumoto et al. 2014; Tiburzy et al. 2014; Fournier et al. 2012; Bermejo et al. 2013). Whether plasmablasts promote islet inflammation through dysregulated production of cytokines is worth further exploration. Secondly, the specificity of plasmablasts was not detected in this study. Identifying islet antigen-specific plasmablasts will help understand the pathogenesis of T1D and improve targeted therapy. Thirdly, there are discrepancies between the mice model and human disease. In patients with T1D, the number of infiltrating cells in islets is small or modest, while a massive lymphocyte infiltration is observed in the NOD mice (Morgan and Richardson 2018). The involvement of plasmablasts in islet destruction should be verified in patients with recent-onset T1D in the future.

## Conclusions

This study suggests a role for plasmablasts in driving effector T cell response against beta cells and promoting the development of T1D. These findings indicates that targeting plasmablasts may offer new strategies for inhibiting T cell activation and limiting islet autoimmunity. Moreover, detection of circulating plasmablasts might provide a new indicator for assessing islet autoimmunity and the progression of T1D.

## Supplementary Information

**Additional file 1: Table S1.** Reagents used for flow cytometry. Figure S1. Flow cytometry gating strategy in mice. **Figure S2.** Features of plasmablasts in patients with new-onset T1D. **Figure S3.** Reconstruction of adoptively transferred cells in the PLNs of recipient mice 4 weeks after transfer. **Figure S4. **The activation of CD8^+^ T cells is not influenced by plasmablasts. **Figure S5. **The number of plasmablasts did not correlate with beta cell function in patients with long-term T1D or T2D. **Figure S6.** Individual staining of CD138, CD4, CD8, and insulin (related to Fig. 4J). **Figure S7.** Individual staining of CD138, CD4, CD8, and insulin (related to Fig. 5C). **Figure S8.** Plasmablasts promote the IFN-γ production by T cells in T1D (answer to the reviewer).

## Data Availability

The data used and/or analyzed during the current study are available from the corresponding author on reasonable request.

## Abbreviations

2hCP: 2-Hour postprandial C-peptide
FCP: Fasting C-peptide
DK: Diabetic ketosis
DKA: Diabetic ketoacidosis
GADA: Glutamate decarboxylase antibody
IA2A: Tyrosine phosphatase antibody
IAA: Insulin antibodies
ICA: Islet cell antibodies
MSC: Mesenchymal stromal cell
NOD mice: Non-obese diabetic mice
PLN: Pancreatic lymph node
PBMC: Peripheral blood mononuclear cell
T1D: Type 1 diabetes
T2D: Type 2 diabetes
