# Supplementary material for: Increased plasmablasts enhance T cell-mediated beta cell destruction and promote the development of type 1 diabetes
Source: Mol Med. 2022 Feb 5;28:18. doi: 10.1186/s10020-022-00447-y (PMC8818172; doi:10.1186/s10020-022-00447-y)
Supplement: Supplementary file 1 — Additional file 1: Table S1. Reagents used for flow cytometry. Figure S1. Flow cytometry gating strategy in mice. Figure S2. Features of plasmablasts in patients with new-onset T1D. Figure S3. Reconstruction of adoptively transferred cells in the PLNs of recipient mice 4 weeks after transfer. Figure S4. The activation of CD8+ T cells is not influenced by plasmablasts. Figure S5. The number of plasmablasts did not correlate with beta cell function in patients with long-term T1D or T2D. Figure S6. Individual staining of CD138, CD4, CD8, and insulin (related to Fig. 4J). Figure S7. Individual staining of CD138, CD4, CD8, and insulin (related to Fig. 5C). Figure S8. Plasmablasts promote the IFN-γ production by T cells in T1D (answer to the reviewer). [file 10020_2022_447_MOESM1_ESM.docx]

Supplementary Materials

# Supplementary Table

**Supplementary Table 1. Reagents Used for Flow Cytometry**

| **Antibodies for human samples** | **Clone** | **μl/test** | **Source** |
| --- | --- | --- | --- |
| Anti-CD19 | SJ25C1 | 1 | BD Biosciences |
| Anti-CD27 | M-T271 | 1 | BD Biosciences |
| Anti-CD38 | HB7 | 1 | BD Biosciences |
| Anti-CD45 | H130 | 1 | BD Biosciences |
| Anti-CD80 | FUN-1 | 1 | BD Biosciences |
| Anti-CD86 | HL307.4 | 1 | BD Biosciences |
| Anti-CD40 | 5C3 | 1 | BD Biosciences |
| Anti-HLA DQ/DR/DP | clone Tu39 | 1 | BD Biosciences |
| **Antibodies for mouse samples** | **Clone** | **μl/test** | **Source** |
| Anti-CD138 | 281-2 | 1 | BD Biosciences |
| Anti-CD44 | IM7 | 1 | BD Biosciences |
| Anti-CD3e | 145-2C11 | 1 | BD Biosciences |
| Anti-CD4 | RM4-5 | 1 | BD Biosciences |
| Anti-CD8 | 53-6.7 | 1 | BD Biosciences |
| Anti-CD25 | PC61 | 2.5 | BioLegend |
| Anti-CD69 | H1.2F3 | 0.625 | BioLegend |
| Anti- IFN-γ | XMG1.2 | 1 | BD Biosciences |
| Anti-TNF-α | MP6-XT22 | 2 | BioLegend |
| Anti-granzyme B | NGZB | 0.625 | eBiosciences |
| Anti-perforin | eBioOMAK-D | 5 | eBiosciences |

Note: Each antibody was diluted according to the manufactures instruction and validated.

# Supplementary Figures

#
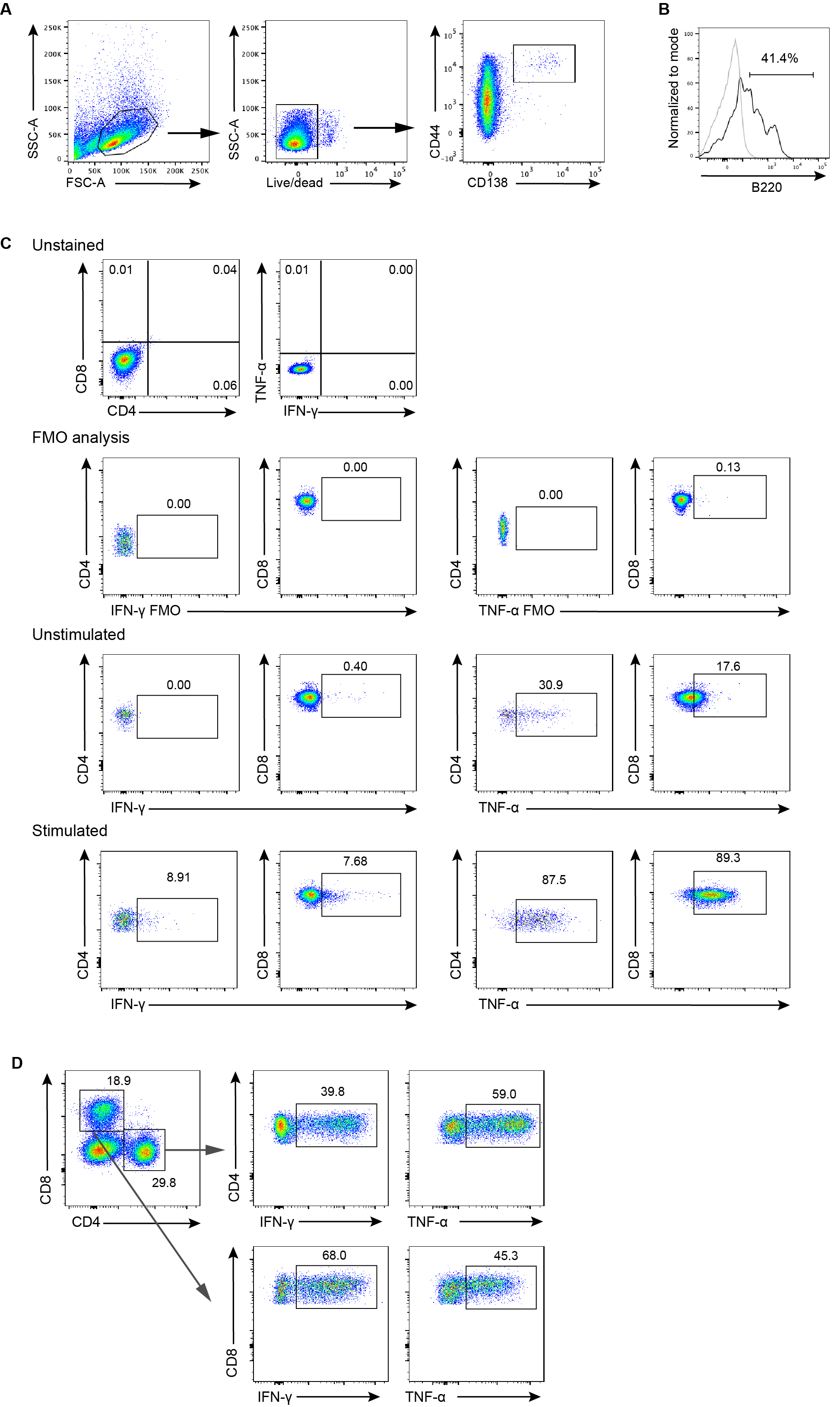


**Supplementary Figure 1. Flow cytometry gating strategy in mice. (A)** Gating strategy of plasmablasts in mice. **(B)** B220 expression on mice plasmablasts. **(C)** Gating strategy of IFN-γ and TNF-α positive T cells in mice. **(D)** Representative cytogram showing staining of IFN-γ and TNF-α in T cells in recipient NOD/SCID mice.

**
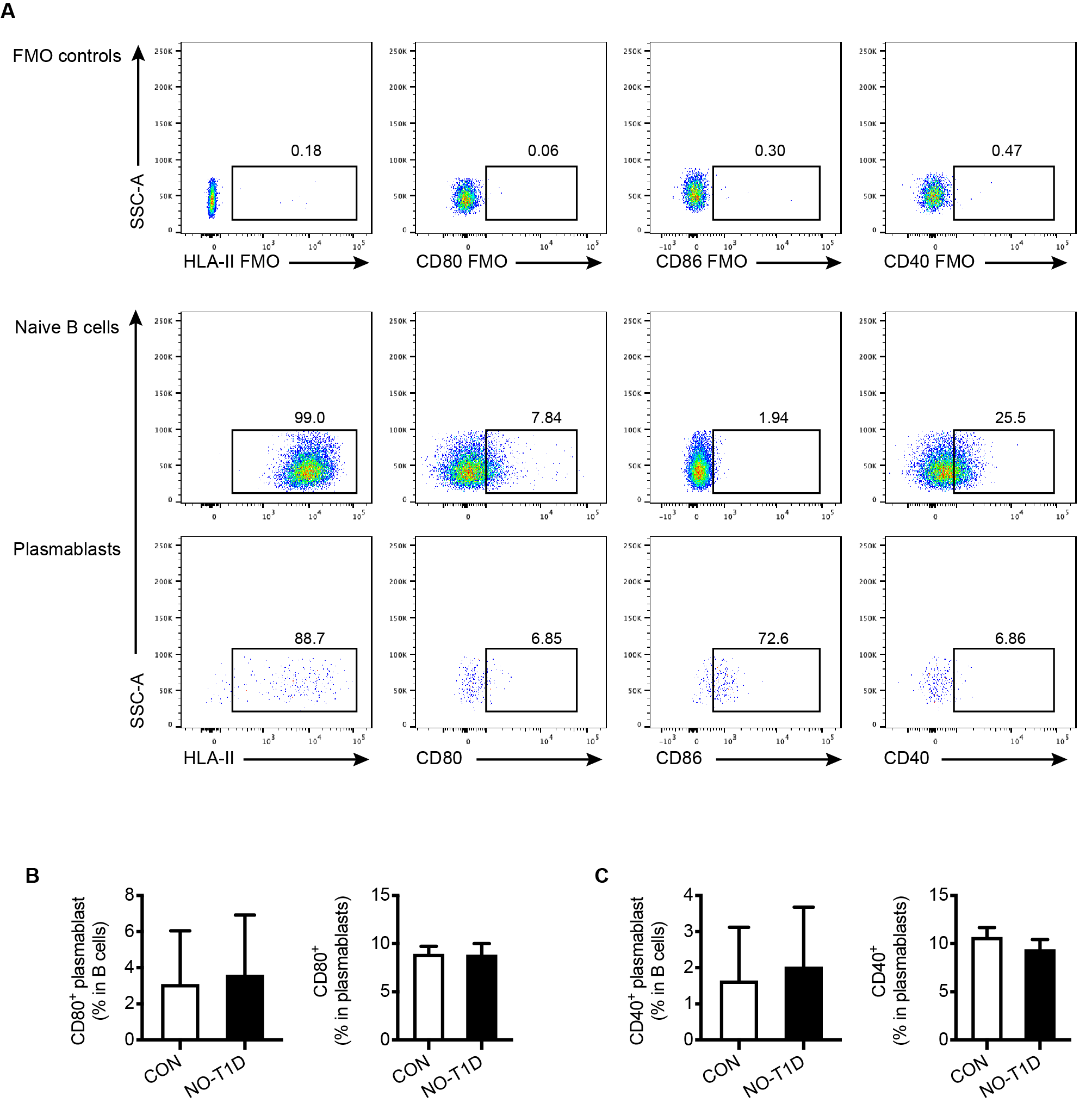
**

**Supplementary Figure 2. Features of plasmablasts in patients with new-onset T1D. (A)** Representative dot plots for MHC-II, CD80, CD86, and CD40 staining. FMO controls were used to help gate the positive cells.  **(B-C)** Comparison of the CD80^+^ and CD40^+^ plasmablast frequencies between control subjects and patients with new-onset T1D. Data indicate mean ± SEM. Independent *t* test.

**Supplementary Figure 3. Reconstruction of adoptively transferred cells in the PLNs of recipient mice 4 weeks after transfer.**

**Supplementary Figure 4. The activation of CD8^+^ T cells is not influenced by plasmablasts. (A)** Comparison of CD8^+^ T cell numbers in the spleens and pancreatic lymph nodes between PB+T group and T group. **(B-C)** Comparison of activated CD8^+^ T cells, as assessed by CD25 and CD69, in the pancreatic lymph nodes (B) and spleens (C) between PB+T group and T group. **(D)** Comparison of IFN-γ^+^, and TNF-α^+^, granzyme B^+^, and perforin^+^CD8^+^ T cells in the pancreatic lymph nodes between PB+T group and T group. Data indicated mean ± SD. Difference were analyzed by Independent *t* test. * *P*<0.05, ** *P*<0.01. **(E)** Flow cytometry analysis of the islet cell apoptosis. **(F)** Apoptosis of islet cells in the indicated conditions.


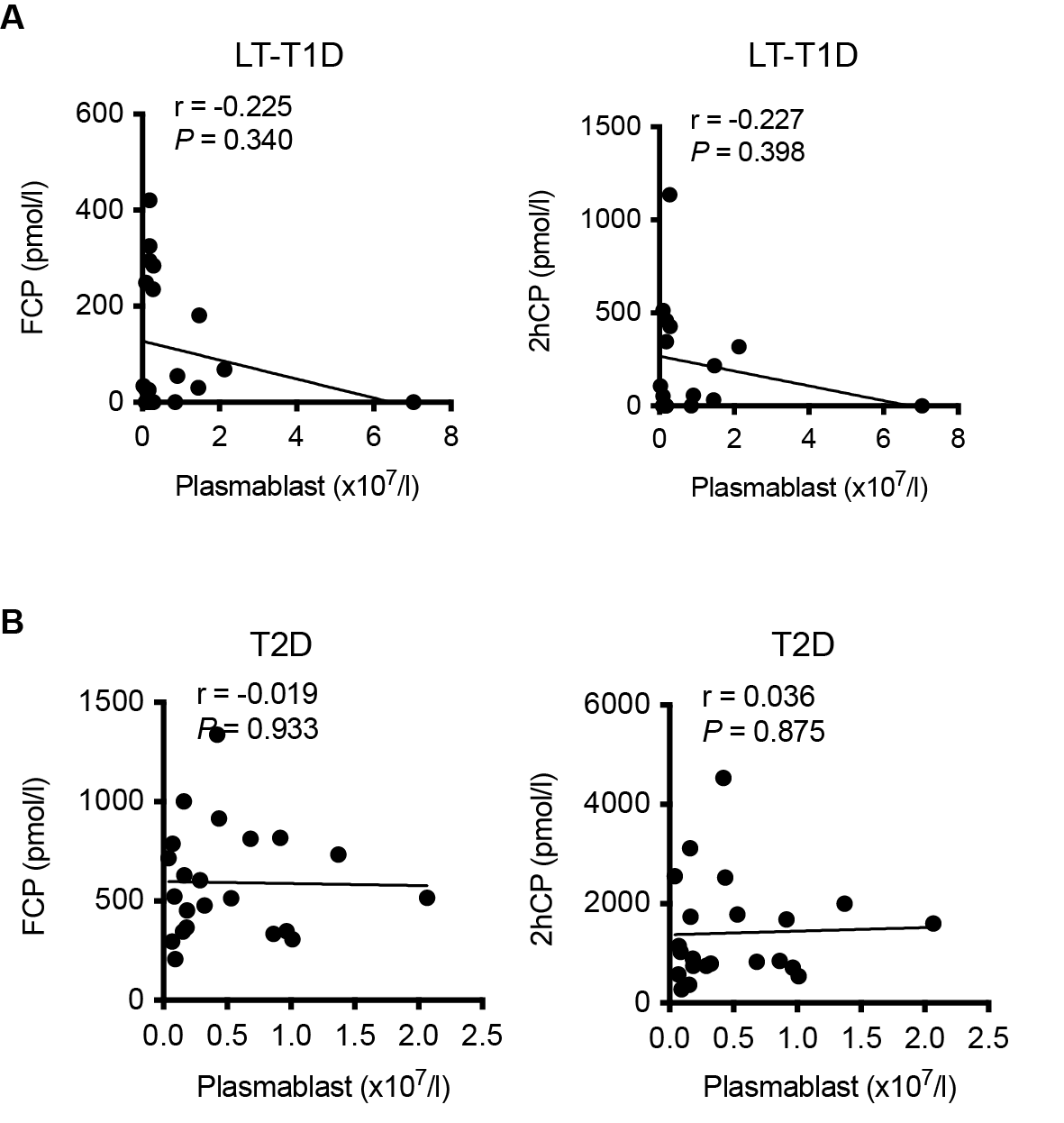


**Supplementary Figure 5. The number of plasmablasts did not correlate with beta cell function in patients with long-term T1D or T2D. (A)** Correlation between the number of plasmablasts and FCP (n=20) and 2hCP (n=16) in patients with long-term T1D. **(B)** Correlation between the number of plasmablasts and FCP (n=22) and 2hCP (n=22) in patients with T2D.


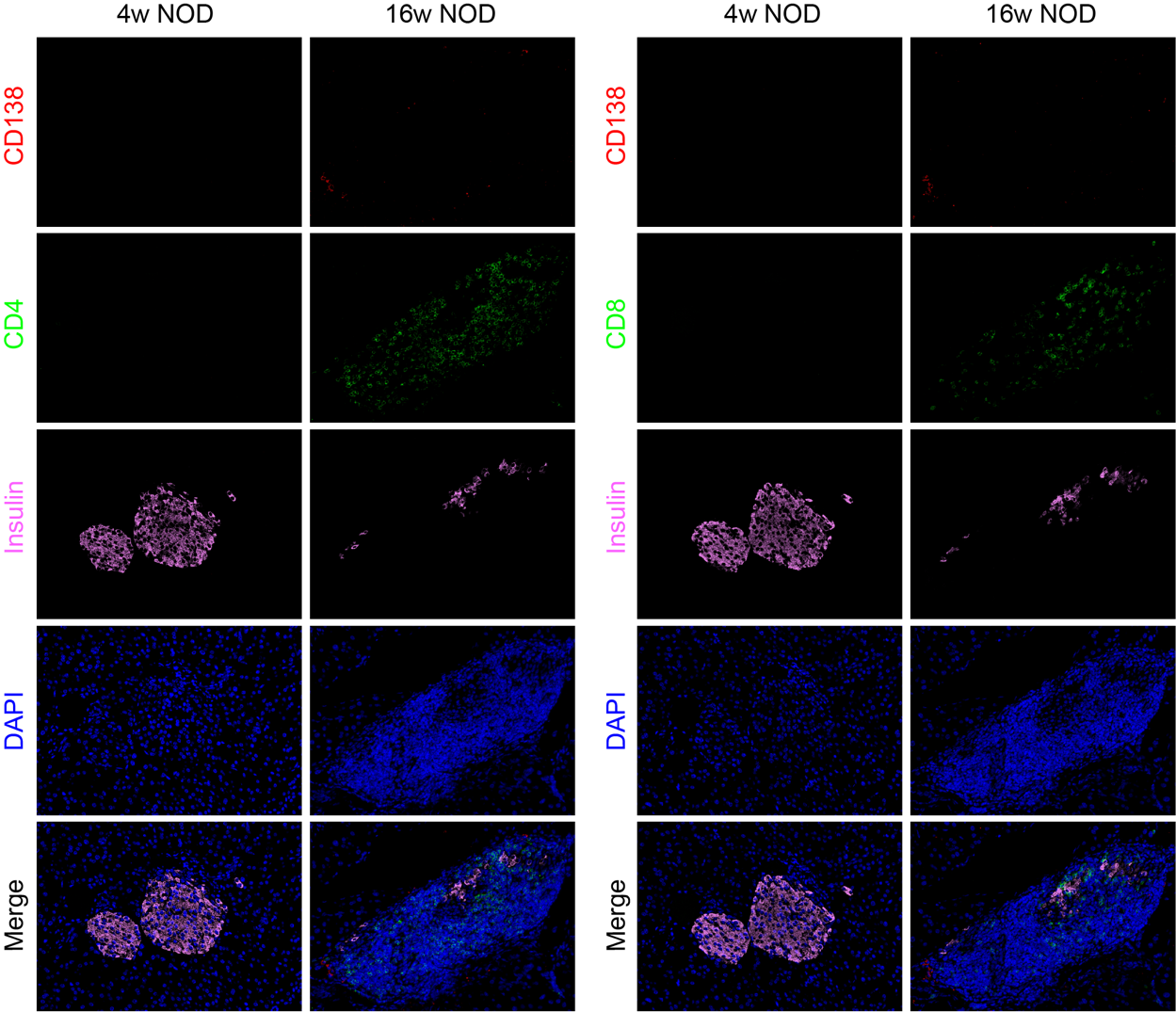


**Supplementary Figure 6. Individual staining of CD138, CD4, CD8, and insulin (related to Figure 4J).**


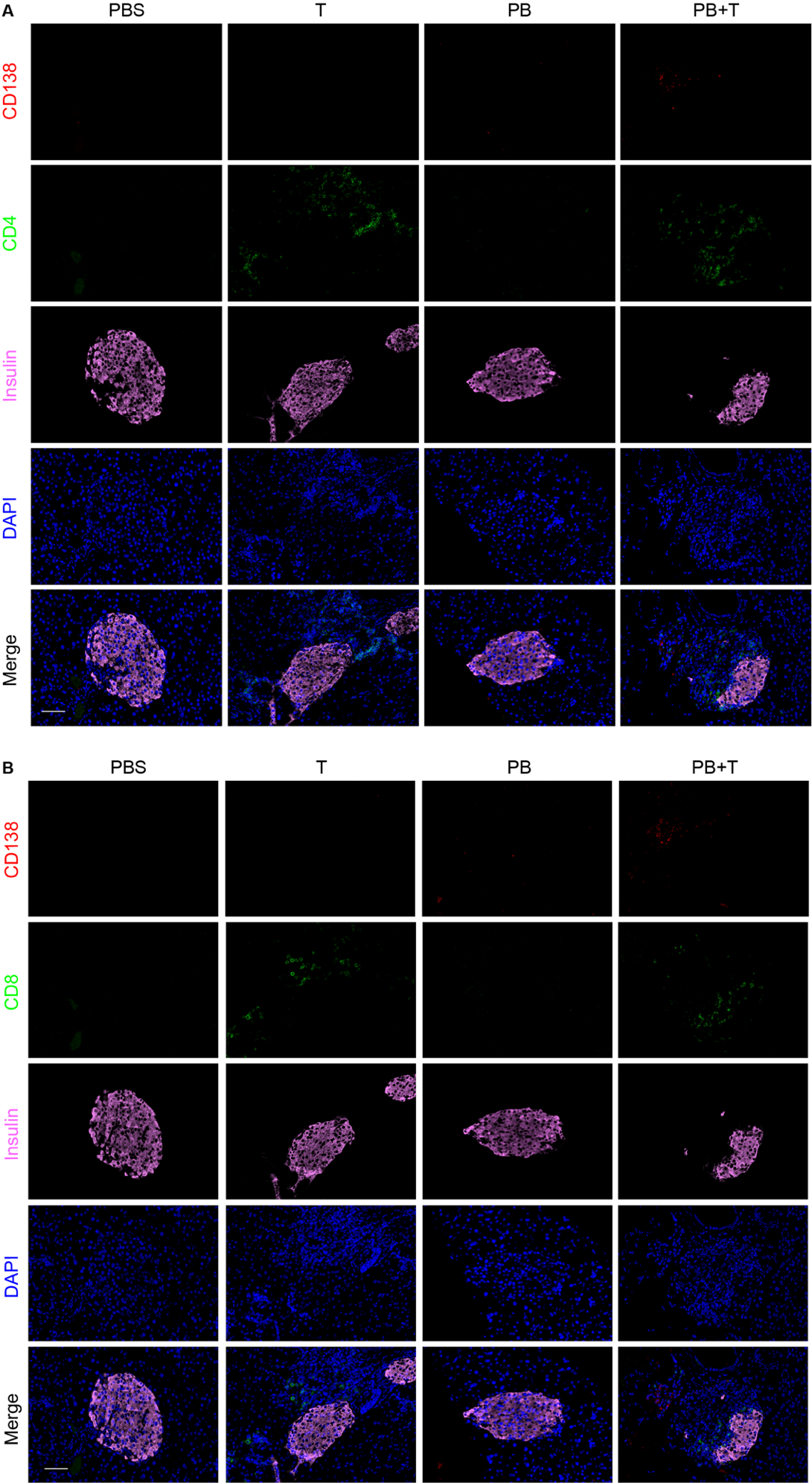


**Supplementary Figure 7. Individual staining of CD138, CD4, CD8, and insulin (related to Figure 5C).**


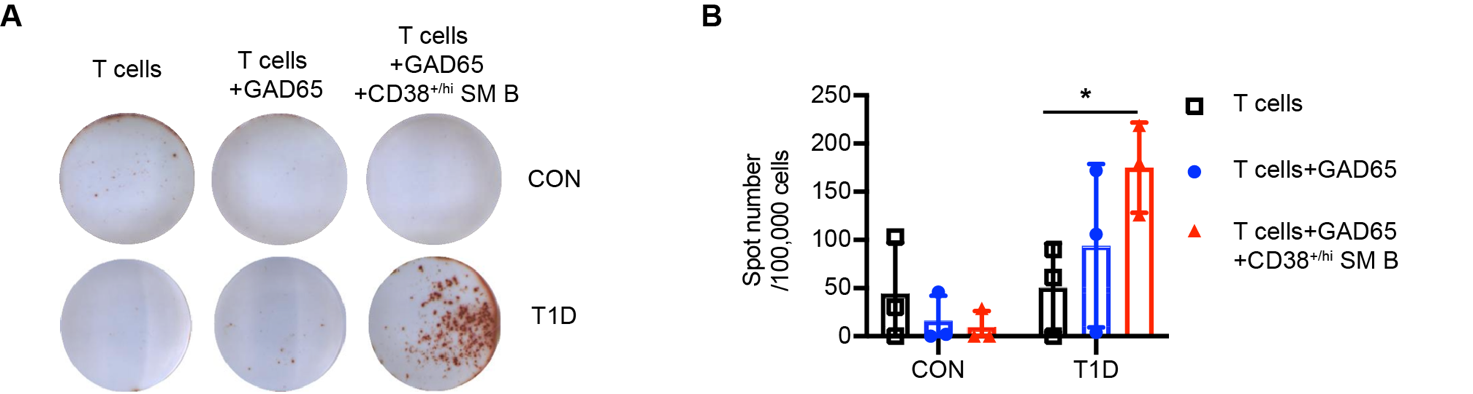


**Supplementary Figure 8. Plasmablasts promote the IFN-γ production by T cells in T1D.** Representative images and data plot of IFN-γ production from patients with T1D or control individuals detected by ELISPOT assay. The plasmablast population co-cultured with T cells contains CD38^+^ SM B cells due to the extremely low amount of plasmablasts in peripheral blood.
